# Why are neurotransmitters neurotoxic? An evolutionary perspective

**DOI:** 10.12688/f1000research.4828.2

**Published:** 2014-12-02

**Authors:** Keith D. Harris, Meital Weiss, Amotz Zahavi

**Affiliations:** 1 Department of Zoology, Tel-Aviv University, Tel Aviv, 69978, Israel; 2 Sagol School of Neuroscience, Tel-Aviv University, Tel Aviv, 69978, Israel

## Abstract

In the CNS, minor changes in the concentration of neurotransmitters such as glutamate or dopamine can lead to neurodegenerative diseases. We present an evolutionary perspective on the function of neurotransmitter toxicity in the CNS. We hypothesize that neurotransmitters are selected because of their toxicity, which serves as a test of neuron quality and facilitates the selection of neuronal pathways. This perspective may offer additional explanations for the reduction of neurotransmitter concentration in the CNS with age, and suggest an additional role for the blood-brain barrier. It may also suggest a connection between the specific toxicity of the neurotransmitters released in a specific region of the CNS, and elucidate their role as chemicals that are optimal for testing the quality of cells in that region.

## Introduction

Some non-peptide chemicals that function as neurotransmitters in the central nervous system (CNS), such as dopamine and serotonin, have toxic effects ^1– 4^. Neurodegeneration can result from the deregulation of the concentration of these neurotransmitters ^5– 7^. It is known that neurotransmitters such as serotonin, acetylcholine (ACh), glutamate and gamma-aminobutyric acid (GABA) function as signals between non neuronal cells in the periphery ^8– 12^, and have evolutionarily conserved roles, serving also as signals in plants ^13, 14^ and unicellular organisms ^15^. This does not necessarily explain their adaptive role as signals in the CNS, as at synapses a variety of less toxic chemicals could have served the same role, had they been loaded into vesicles in the pre-synaptic neuron and had complementary receptors on the post-synaptic neuron. In the following we attempt to highlight the potential insights that may arise from applying the theory of signal selection ^16^ to the evolution of signals between cells in multicellular organisms. The theory of signal selection, based on the handicap principle, suggests that the properties of the signal serve as a test of the information encoded in the signal. The theory revolutionized the study of signaling between organisms ^17, 18^. The application of the theory to the evolution of neurotransmitters suggests that neurotransmitters are selected in part because of their toxicity, which serves as a test of the quality of the releasing cell and its connectivity with neighboring cells, and facilitates the selection of neuronal pathways.

## The theory of signal selection

The theory of signal selection was developed by Zahavi ^19, 20^ to explain why peahens are stimulated by a trait that imposes a handicap on the male, rather than paying attention to more positive traits in the males that court them. Zahavi suggested that peahens are attracted by peacocks that carry the burden of a long and heavy tail because this burden constitutes a handicap that tests the quality of the displaying peacock. This interpretation pointed at the objective information provided by the signal, which results in the peahen responding to one peacock and rejecting others; it is not coincidental that peahens are attracted to males with heavy tails, rather, it is the tested and reliable information provided by the cumbersome tail that selected for the interest of the female in the level of the handicap imposed on the male by its tail.

We suggest that, similarly to the burden imposed by the peacock’s tail, a signal’s toxicity is necessary to impose a specific chemical burden on the signaling cell to ensure that the signal inherently provides reliable information on some properties of the signaling cell. It is reasonable to assume that if signals within multicellular organisms were consensus signals that did not inherently correlate to a specific metabolic activity of the signaling cell, a larger variety of chemicals could have been selected as signals within multicellular organisms. In addition, phenotypes which had not developed to signal could signal in error, while the level of the signal could misrepresent the metabolic state of the signaler. We suggest that the investment in reliable signaling in multicellular organisms is necessary in order to reduce the potential harm of such errors ^16^. Tests must be difficult in order to provide meaningful and reliable results ^16^, and hence we expect that, if neurotransmitters also test the quality of the releasing cell, they should be directly toxic in a way that tests the message encoded in the signal.

## Neurotransmitter toxicity and its implication in neurodegeneration

In the CNS, neurotransmitters play a central role in relaying information at chemical synapses. This role involves their vesicular secretion by the pre-synaptic cell and interaction with receptors on the post-synaptic cell. However, neurotransmitters are also released outside synapses in high concentrations prior to blood-brain barrier development ^21, 22^ and as part of non-synaptic forms of intercellular communication in the mature brain ^23^. Synaptic transmission requires the rapid clearance of the secreted or released neurotransmitter via uptake by neurons and astrocytes ^24^. When these mechanisms are deregulated, the accumulation of neurotransmitter in the extracellular matrix can lead to neurodegeneration ^5– 7^. Here we review briefly the toxicity of some neurotransmitters and its role in neurodegeneration.

## Glutamate

Glutamate exerts neurotoxicity via excitotoxicity caused by the overactivation of NMDA receptors ^25^ and oxidative toxicity caused by the inhibition of cysteine uptake via uptake by the cysteine-glutamate anti-porter ^26^. As glutamate uptake is an energy-dependent process that involves the co-transport of sodium ^27^, glutamate uptake is reversed in hypoxic conditions and leads to an increase in extracellular glutamate ^28^. The increase of extracellular glutamate has been implicated as a causative factor in numerous pathologies, including stroke ^29^, Huntington’s disease, Parkinson’s disease and amyotrophic lateral sclerosis ^30^.

Despite its abundance, glutamate is stored mostly in subcellular compartments ^31^: in astrocytes its uptake is coupled with its conversion to glutamine ^32^ and in neurons the synthesis of glutamate from 2-oxoglutarate ^33^ or glutamine ^32^ is correlated to its uptake into vesicles, suggesting that it is also potentially toxic within the cytoplasm. In addition to glutamate toxicity that is mediated by its interaction with receptors and secondary to its uptake mechanisms, evidence of the interaction of glutamate with oxygen radicals could point to potential direct damage of glutamate to membranes. In the presence of hydroxyl radicals and molecular oxygen, glutamate is oxidized to 2-oxoglutarate in a reaction that releases hydrogen peroxide ^34, 35^. Glutamate in particular has a relatively high yield of peroxide in the presence of oxygen radicals, relative to glutamine, glycine and aspartate ^34^. This process is also iron-dependent, the presence of which is a causative factor of neurodegeneration involving radical oxygen species ^36^.

## Dopamine

Dopamine is involved in the pathogenesis of Parkinson’s disease, which involves the degeneration of dopaminergic neurons in the substantia nigra, leading to motor dysfunction ^5, 6^. The loss of dopaminergic neurons has been linked to dopamine’s cytotoxicity that results from the deregulation of its metabolism in these neurons ^6^.

Dopamine is directly toxic in its oxidized semiquinone and quinone forms ^1, 37^. Dopamine toxicity is also related to the presence of metal ions such as iron ^4^, which increase its oxidation to neurotoxic metabolites ^38^, while metal ion chelators have a protective effect in Parkinson’s disease ^39^. It has already been suggested that redox mechanisms that render intracellular dopamine toxic in the cytosol could also render extracellular dopamine toxic ^3^.

## Serotonin

Serotonin is sensitive to oxygen radicals, and its indole moiety is readily oxidized in the presence of hydroxyl radicals to form neurotoxic metabolites of serotonin ^1^. The indole moiety of serotonin can undergo oxidation by indoleamine 2,3-dioxygenase to form kynurenine, which can be metabolized further into various neurotoxic chemicals ^40^. This pathway of serotonin metabolism has been implicated in neurodegeneration associated with depression ^41^. Serotonin is toxic in the presence of copper ^42^, causing intracellular damage such as DNA strand cleavage ^43^. Serotonin is also toxic in the presence of iron ^2^, causing mitochondrial damage ^44^. This suggests a role for serotonin in copper and iron mediated neurodegeneration.

Serotonin can also interact with lipid membranes ^45^, partially intercalating into the phospholipid layer and thus causing structural changes in the membrane. It has been shown that the interaction of neurotransmitters with the cell membrane can have a non-specific anesthetic effect on receptor activity ^46^, and so chronic exposure to serotonin may alter membranal homeostasis.

## Acetylcholine

As far as we are aware, there is currently no experimental evidence of direct ACh toxicity. However, the overstimulation of ACh receptors as a result of ACh accumulation that is caused by acetylcholinesterase inhibition can lead to cholinergic toxicity ^47, 48^. This toxicity may involve the release of choline from phosphatidylcholine that is downstream of muscarinic ACh receptors ^49^, leading to phosphatidylcholine depletion. In addition, the use of nicotinic ACh receptor antagonists has shown to reduce the neurotoxicity of the Alzheimer’s disease-related peptide, β-amyloid ^50^.

ACh interacts with lipid bilayers and elicits changes in the organization of the lipid bilayer ^51^. This interaction is non-specific, slower than receptor activation, and has a longer duration ^46^. We speculate that the accumulation of ACh could interfere with the membrane morphology ^46^ and consequently may interfere with its function.

## Testing neurotransmitter toxicity

Though the putative toxicity of neurotransmitters presented above suggests that our hypothesis can be generalized to most neurotransmitters, only a small number of neurotransmitters have been shown to have direct toxicity. In order to test the direct toxicity of a neurotransmitter, it is necessary to create a cell line that does not express adaptive defense mechanisms (such as specific receptors or degrading enzymes that bind the neurotransmitter), and expose it to varying concentrations of the neurotransmitter. If the concentration of the neurotransmitter in the medium has no effect on the viability of the cells, then it is reasonable to assume that the neurotransmitter is not directly toxic. This type of experiment could test our hypothesis.

## The function of neurotransmitters in the brain – some considerations resulting from our evolutionary perspective

The consideration of a function for neurotransmitters as a reliable representation of the specific activity of the releasing cell, rather than simply as chemicals that facilitate the transfer of information between neurons, may contribute novel deliberations and interpretations of known phenomena.

The formation of connections between neurons in the vertebrate CNS during embryogenesis and development is a dynamic process in which neurons that do not form synapses are eliminated ^52, 53^, while neurons forming new synapses survive into adulthood ^54, 55^. In addition, since neurons have an array of potential connections, a selection process is involved in the development and ongoing activity of neuronal networks ^52, 55– 57^. Hence, we suggest that the toxic neurotransmitters that are released from neurons in the CNS function as tests of neuronal quality. The toxicity is important for the process of selection that is involved the selection of the optimal pathways for relaying information between and within specialized CNS centers.

A better reflection of quality is obtained when tested in more than one parameter. In the choice of mates, birds display their quality through several signals such as dancing, colors and vocalizations ^16^. This may be also the reason why more than one neurotransmitter participates in the selection of neuronal connections. Indeed, most synapses depend on more than one neurotransmitter in order to function ^58^.

Several observations support the notion of the importance of neurotransmitters in the selection of synapses: glutamate signaling in the auditory system is essential for the normal development of inhibitory circuits, in which some synapses are strengthened and others are silenced ^57^. Glutamate is also important in the maturation of neuronal pathways in the mushroom bodies of *Drosophila* through non-synaptic mechanisms ^59^. GABA is similarly involved in the development of neuronal circuits through non-synaptic mechanisms ^60^.

### Brain centers and their specific composition of neurotransmitters

If, as we suggest, released neurotransmitters represent the phenotypic qualities of the releasing cell, the fact that specialized CNS centers release a specific combination of neurotransmitters implies that the neurons in these centers have distinct metabolic activities that relate to the function of the center. For example, in the raphe nuclei, the main source of serotonin in the brain, there is a high extracellular concentration of serotonin, the source of which is a non-synaptic release which is correlated with the activity level of the raphe nuclei ^61^. We suggest that the release of serotonin was adopted, and still functions as, a paracrine signal between cells in the raphe nuclei that facilitates, by a selection process, a local synchronization of activity.

Neurons within a specialized population of cells vary in their morphology, their proximity to the sources of metabolites or to incoming stimuli from outside the center, and may vary also with many other parameters ^62^. The specific neurons that are phenotypically more capable to carry out their function are those that react to and process the information received in the center, defining the output of the center. For instance, soma size determines electrophysiological differences between neurons of retinal ganglions, larger neurons having greater excitability ^63^.

It is reasonable to assume that these phenotypic differences that relate to metabolite capability also determine the level of neurotransmitter released by neurons in the ganglion: less active phenotypes cannot counter the toxic effects of the serotonin released by the more active phenotypes, and consequently lower their metabolism in order to reduce the concentration of serotonin around their outer membrane. Indeed, the release of serotonin in the raphe nuclei is reduced by an increase in its extracellular concentration ^61^, which, we suggest, is a consequence of reduced activity in neurons that reduce their release. If serotonin was not toxic, the more active phenotypes, which produce and release higher concentrations of serotonin, would not reduce the synthesis of serotonin in less active phenotypes, and serotonin release could not serve as a mechanism of selection.

Furthermore we speculate that if the activity of a specific brain center entails the production of a particular waste product, this waste may serve at synapses as an optimal neurotransmitter to ensure that the information provided by the electrical stimulus originates in a specific center.

### Phylogeny and neurotransmitters

 Glutamate serves as the primary excitatory neurotransmitter at the insect neuromuscular junction ^64, 65^, whereas in mammals acetylcholine serves this role. The choice of neurotransmitter could be explained by the fundamental anatomical and physiological difference between mammals and insects: while insects receive oxygen directly to cells via trachea, and thus avoid contact between the extracellular medium and oxygen radicals, mammals receive oxygen through the extracellular medium. In other words, the insect neuromuscular junction is not exposed to oxygen to the same degree as the mammalian neuromuscular junction, therefore, glutamate is not exposed to oxidation and can be used as a neurotransmitter without having the same level of toxicity as in mammals. As a consequence of the ability to explain neurotransmitter choice based on anatomical and physiological differences, we did not place emphasis on the phylogenetic context to explain the usage of a particular neurotransmitter for its function.

### The blood-brain barrier

The blood-brain barrier of vertebrates separates the extracellular environment of neurons in the CNS from changes caused in peripheral tissues ^66^. It has been suggested that the blood-brain barrier facilitates the maintenance of the highly regulated microenvironment of the synapse by preventing neurotransmitters synthesized in the periphery from reaching synapses in the CNS, creating a “cross-talk” between peripheral and neuronal signaling ^67^. We suggest, in addition, that if neurotransmitters test and therefore represent the metabolic activity of neurons, then any influx of neurotransmitters from the periphery into the CNS could potentially interfere with that function. In other words, the extracellular concentration of neurotransmitters can only reliably reflect the metabolism of neurons if it is isolated from neurotransmitters produced in the periphery. This may constitute an additional adaptive significance for the mechanisms that prevent toxic neurotransmitters from diffusing through the blood-brain barrier.

### Reduction of neurotransmitters in the aging brain

Aging is accompanied by changes in neurotransmitter concentrations in the brain, and in a number of regions there is a significant decrease in the concentration of glutamate, dopamine and serotonin ^68– 71^. It is possible to interpret the depletion of certain neurotransmitters in old age as an adaptive response to the reduced ability of aging cells to counter the toxicity of these neurotransmitters. Under such conditions it is preferable to reduce the severity of the test rather than to forgo the test altogether. Indeed, dopamine synthesis is regulated by the redox state of the cell, and oxidative stress leads to an inhibition of tyrosine hydroxylase, the rate-limiting enzyme in the synthesis of dopamine ^72, 73^. This might explain why restoring the toxicity through an increase in the concentration of certain neurotransmitters, in cells that cannot counter this toxicity, may cause long-term damage, as in the case of l-DOPA treatment for Parkinson’s disease ^74^, while treatment with anti-oxidants has the potential to restore neurotransmitter concentrations to normal levels ^75^.

### The evolution of chemical signaling in the brain

It has already been suggested by Le-Corronc *et al.* ^76^ that the developmental role of neurotransmitters as paracrine signals precedes their role as facilitators of synaptic transmission. Our evolutionary perspective suggests that neurotransmitters that functioned in the periphery as paracrine signals, released directly from the cytoplasm, were initially adopted by the CNS to serve as paracrine signals within specialized CNS centers. The toxicity of the neurotransmitters facilitated the selection of the optimal cells for the particular function of the CNS and coordinated the activity of cells within specific CNS centers. The use of these neurotransmitters at synaptic contacts was later adopted as a signature that identifies the origin of the electrical stimulus arriving at the post synaptic neuron, and prevents other electrical stimuli from interfering with the stimuli from the pre-synaptic neuron.

We hope that further studies of the function of a CNS center in relation to its particular metabolism involved in processing information may lead to a greater understanding of the relationship between the activity of neurons within the center, and the specific composition of the neurotransmitters they release.

## An evolutionary model of the stages that selected toxic chemicals as signals

Our evolutionary perspective suggests that toxic waste released into the extracellular environment by the signaling cell, a release that is inherently correlated to the activity of the signaling cell, forces neighboring cells to react to counter the toxicity of the release. Their reaction may provide them with information that can contribute to the coordination of their activity with neighboring cells. Here we explain the model in the context of various examples that were instructive in its development.

Different metabolic activities result in the production of particular waste products. For example, oxidative phosphorylation in mitochondria leads inevitably to the production of reactive oxygen species ^77^. Another example is the release of ACh, which is correlated to calcium influxes ^78^: as motor activities require the influx of calcium ion into the cytoplasm ^79^, and as ACh is also a positive ion, its release is an inevitable result of the influx of calcium ions ^78^. While other positive ions may be released as a result of the influx of calcium, ACh is quickly hydrolyzed outside the cell ^80^, as opposed to inorganic ions, and therefore reliably reflects in more detail than other ions the current activity of the releasing cell.

It is also reasonable to assume that the level of the waste released is correlated to the level of the activity of the releasing cell, such as the correlation between carbon dioxide production and the level of respiration ^81^.

Among the waste products released, some are more toxic and potentially harmful to nearby cells, since waste released within a multicellular organism encounters the outer cell membrane of nearby cells in addition to its potential harm to the signaling cell.

Cells exposed to a toxic chemical must counter the toxicity via (1) producing and releasing anti-oxidants, such as the release of ascorbate to reduce dopamine-mediated oxidative damage ^37^, (2) degrading the chemical enzymatically, such as acetylcholinesterase ^80^, or (3) transporting the chemical into the cytoplasm where it can be converted into less harmful chemicals or transported into and stored inside vesicles, as in the case of glutamate and dopamine ^5, 24^.

The uptake of glutamate or the release of antioxidants which counters the toxicity of dopamine is correlated to their respective concentrations outside the cell. The response to a toxic chemical must be related to its concentration if it is to counter its toxicity. In addition, the toxicity also harms the membrane of the releasing cell, limiting its metabolic activity in order to prevent the cell from increasing the level of release beyond its ability to cope with the toxicity, as evidenced by the inhibition of serotonin secretion and synthesis by extracellular serotonin ^61^.

Consequently, the activity of a cell to counter the toxicity of chemicals in its extracellular environment can provide it with information on its potential to be active as compared with that of the secreting cells. Such information can serve as a cue to facilitate the coordination of activities with those of the releasing cell, for instance, in the course of the development of osteoblasts that is mediated by glutamate ^82^, to either differentiate, undergo mitosis or apoptosis. Coordination between neighboring cells is necessary within multicellular organisms, and we suggest that the information provided by the reaction to released toxic waste can facilitate this coordination: for instance, in airway epithelium, which coordinates cilia beating via ACh 7 ^83^, or in developing tissues such as developing osteoblasts, which coordinate development via glutamate signaling ^84^.

Before the organism benefited from the reaction of neighboring cells to the release of the toxic chemical, mutations that resulted in increased synthesis of the released toxic chemical would have been detrimental. However, once neighboring cells became attentive to changes in the level of the released chemical, the organism could benefit from enzymes that increase the production of the toxic chemical in the releasing cell, which can provide more detailed and accurate information about a change in its metabolism, and facilitate the synchronization of activities between neighboring cells.

This extra investment in increasing the production of a toxic chemical (the handicap), changes the released chemical from a cue into a signal, and provides the basis for a paracrine signaling system ^16, 85^. We follow Maynard Smith and Harper ^18^ in defining a signal as a trait that benefits the signaler only if the receiver reacts to it in a way that benefits the signaler.

It is interesting to note that the CNS uses ACh to stimulate peripheral cells, which is the same signal that is used in the periphery in paracrine signaling, rather than evolving a novel neurotransmitter, a process that would require the coevolution of receptors and complementary transduction systems to process the information. It is possible that the release of ACh from myocytes ^86^, which we suggest is an inevitable result of calcium influx, can serve as a paracrine signal and as a retrograde signal that provides reliable information regarding myocyte contraction to extrasynaptic ACh receptors on the motor neuron ^85^. It is possible that other neurotransmitters also serve as retrograde signals. For example, glutamate serves as a retrograde signal between cerebellar Purkinje neurons ^87^.
