# Supplementary material for: Why are neurotransmitters neurotoxic? An evolutionary perspective
Source: F1000Res. 2014 Dec 2;3:179. Originally published 2014 Jul 30. [Version 2] doi: 10.12688/f1000research.4828.2 (PMC4288432; doi:10.12688/f1000research.4828.2)
Supplement: Peer review history

# Peer review: Referee response for version 2

**DOI:** 10.5256/f1000research.6227.r7121

**Published:** 2014-12-22

**Authors:** Ulrich Technau

**Affiliations:** 1 Department for Molecular Evolution and Development, University of Vienna, Vienna, Austria

The authors do not include a phylogenetic perspective of their evolutionary scenario by arguing that for instance insects use glutamate for neuromuscular junctions, while mammals use acetylcholine. Of course neurotransmitters can be co-opted to other types of neurons and contexts, but my point was different: from the existing knowledge, it seems that nervous systems evolved only once (or twice, if the authors of the Ctenophore genome are right) in animals and the conserved use of the various neurotransmitters is part of that argument. If neurotransmitters evolved as  signals of their state by secreting toxic metabolic waste products, this should also be the case in animals lacking a nervous system, such as placozoans and sponges. I therefore expected a discussion of the potential role of these compounds in these animals.

I have read this submission. I believe that I have an appropriate level of expertise to confirm that it is of an acceptable scientific standard.

# Response

**Published:** 2014-12-22

**Authors:** Keith Harris

**Affiliations:** Tel Aviv University, Israel

We take the evidence of neurotransmitter chemicals serving in signaling roles outside the central nervous system in animals that have a central nervous system as paracrine signals, or in unicellular organisms or plants, as evidence that the signaling role of these chemicals preceded their role in central nervous systems. We have also argued this in a previous paper [85]. We cited in the introduction the work of Csaba [15] in Tetrahymena which suggests that unicellular organisms have hormonal systems which use the same chemicals that serve as hormones and neurotransmitters in animals such as serotonin and insulin.

We would argue that the example of the specialization of neurotransmitters and hormones in insects demonstrates how the physiological context of the signaling molecule selects the signaling molecule rather than a phylogenetic constraint.

# Peer review: Referee response for version 2

**DOI:** 10.5256/f1000research.6227.r6919

**Published:** 2014-12-08

**Authors:** Rony Paz

**Affiliations:** 1 Department of Neurobiology, Weizmann Institute of Science, Rehovot, Israel

It is an interesting perspective. I believe it would be hard to find conclusive evidence to support it, but I think it provides an additional view on the matter.

I have read this submission. I believe that I have an appropriate level of expertise to confirm that it is of an acceptable scientific standard.

# Peer review: Referee response for version 1

**DOI:** 10.5256/f1000research.5153.r6188

**Published:** 2014-09-30

**Authors:** Rony Paz

**Affiliations:** 1 Department of Neurobiology, Weizmann Institute of Science, Rehovot, Israel

This is an interesting speculative paper, suggesting a novel explanation for a long-standing question: why are neurotrasmitters toxic? It applies a similar logic and rationale as in the original handicap-principle (and the extended signal selection) to neurotransmitters and their use as signalling system between neurons. As such, it suggests a nice explanation and the authors supply several examples that this approach can help explain. Yet it also suffers from the lack of more conclusive evidence, as many other evolutionary-driven explanations. I would ask the authors to suggest direct predictions that can be tested in an experimental setup, and supply few examples of putative results that might argue against their idea. If such predictions are provided, the paper will be strengthened and would constitute an important idea.

I have read this submission. I believe that I have an appropriate level of expertise to confirm that it is of an acceptable scientific standard, however I have significant reservations, as outlined above.

# Response

**Published:** 2014-11-21

**Authors:** Keith Harris

**Affiliations:** Tel Aviv University, Israel

We have added in the new revision a suggestion of how our hypothesis might be tested fairly simply, and what results would argue against our idea.

# Peer review: Referee response for version 1

**DOI:** 10.5256/f1000research.5153.r6204

**Published:** 2014-09-22

**Authors:** Ulrich Technau

**Affiliations:** 1 Department for Molecular Evolution and Development, University of Vienna, Vienna, Austria

This paper proposes a somewhat provocative but also inspiring hypothesis which claims that neurotransmitters evolved from a similar principle as the sexual signals in birds: it tests the activity and the status of the signaling cells by secreting a toxic substance. They provide an overview of the toxicity of neurotransmitters when concentrations are slightly unbalanced.

They also propose that the blood-brain barrier evolved as part of the distinct signaling of neurons in the CNS, without disturbance by signals produced in the periphery. The authors provide interesting thoughts as to why and how the transmitter system could have evolved from the release of a toxic waste. This is all fine, but what I miss is the evolutionary perspective promised in the title, which not only is based on a "Gedankenexperiment" but on available evidence. All animals except sponges and placozoans have neurons. Current evidence suggest that neurons from cnidarians and bilaterians have a common origin, which is also reflected by the use of the same transmitters (although the Hydra genome shows that several crucial genes of Ach production are missing). The recent analysis of the ctenophore genome led, however, to the conclusion that neurons evolved independently in ctenophores and bilaterians. Sponges, on the other hand, have many synaptic genes present in the genome, yet lack neurons. In summary, interesting hypothesis, but I miss a discussion of all these available genomic data in the context of the hypothesis.

I have read this submission. I believe that I have an appropriate level of expertise to confirm that it is of an acceptable scientific standard, however I have significant reservations, as outlined above.
